# Distinct Region- and Time-Dependent Functional Cortical Adaptations in C57BL/6J Mice after Short and Prolonged Alcohol Drinking

**DOI:** 10.1523/ENEURO.0077-20.2020

**Published:** 2020-06-05

**Authors:** Reginald Cannady, Sudarat Nimitvilai-Roberts, Sarah D. Jennings, John J. Woodward, Patrick J. Mulholland

**Affiliations:** Department of Neuroscience, Charleston Alcohol Research Center, Medical University of South Carolina, Charleston, SC 29425

**Keywords:** alcohol use disorder, anterior cingulate cortex, ethanol drinking, orbitofrontal cortex, plasticity

## Abstract

Alcohol (ethanol) use disorder is associated with changes in frontal cortical areas including the anterior cingulate cortex (ACC) and orbitofrontal cortex (OFC) that contribute to cognitive deficits, uncontrolled drinking, and relapse. Acute ethanol exposure reduces intrinsic excitability of lateral OFC (lOFC) neurons, while chronic exposure and long-term drinking influence plasticity of intrinsic excitability and function of glutamatergic synapses. However, the time course that these adaptations occur across a history of ethanol drinking is unknown. The current study examined whether short-term and long-term voluntary ethanol consumption using an intermittent access paradigm would alter the biophysical properties of deep-layer pyramidal neurons in the ACC and lOFC. Neuronal spiking varied in the ACC with an initial increase in evoked firing after 1 d of drinking followed by a decrease in firing in mice that consumed ethanol for one week. No difference in lOFC spike number was observed between water controls and 1-d ethanol drinking mice, but mice that consumed ethanol for one week or more showed a significant increase in evoked firing. Voluntary ethanol drinking for 4 weeks also produced a total loss of ethanol inhibition of lOFC neurons. There was no effect of drinking on excitatory or inhibitory synaptic events in ACC or lOFC neurons across all time points in this model. Overall, these results demonstrate that voluntary drinking alters neuronal excitability in the ACC and lOFC in distinct ways and on a different time scale that may contribute to the impairment of prefrontal cortex-dependent behaviors observed in individuals with alcohol use disorder (AUD).

## Visual Abstract

**Figure:**
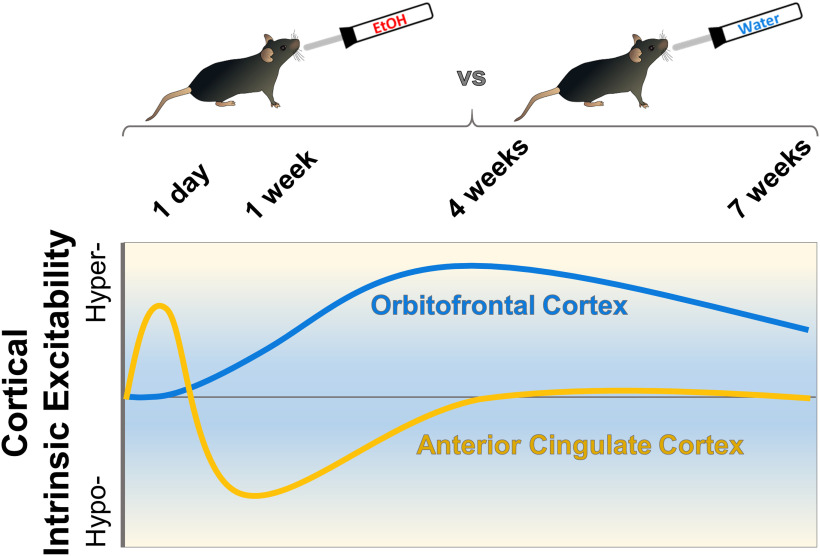

## Significance Statement

Adaptations in function of prefrontal cortex neurons caused by chronic ethanol exposure have been described previously, but the time course and region specificity that these changes occur is unknown. We find that voluntary ethanol drinking in mice produces distinct time-dependent changes in cellular excitability across two cortical subregions that varied by direction and duration. These drinking-induced changes in cellular excitability were specific to action potential firing but not to function of excitatory or inhibitory synapses. Our findings highlight the importance and sensitivity of alterations in cellular firing of cortical neurons that occur early in a drinking history and persist during long-term ethanol consumption.

## Introduction

The prefrontal cortex (PFC) is among several brain regions that exhibit vulnerability to alcohol (ethanol) and is of significance due to its role in cognition (Fuster, 2001). Prolonged drinking produces cognitive deficits that impede recovery efforts due to behavioral inflexibility and impulsivity in individuals with alcohol use disorder (AUD; Pitel et al., 2009; Stavro et al., 2013). Interestingly, PFC-associated cognitive dysfunction is most prominent following early abstinence from chronic ethanol (Loeber et al., 2009; Stavro et al., 2013), suggesting that a focus on PFC plasticity during early withdrawal is clinically relevant. As consumption and ethanol-related deaths increase (White et al., 2020), it is imperative to elucidate the mechanisms underlying uncontrolled drinking.

In preclinical mouse models, chronic ethanol exposure induces deficits in PFC-dependent cognitive tasks (Badanich et al., 2011; Kroener et al., 2012; Salling et al., 2018), alters plasticity of intrinsic excitability and function of excitatory and inhibitory synapses in PFC neurons (Cannady et al., 2018; Renteria et al., 2018; Salling et al., 2018), and produces morphologic adaptations in dendrites and dendritic spines (Kroener et al., 2012; McGuier et al., 2015; McGuier et al., 2018). The orbitofrontal cortex (OFC) and anterior cingulate cortex (ACC) are two cortical regions involved in cognition that have been implicated in ethanol addiction. Studies show that lateral OFC (lOFC) lesions impair reversal learning (Dias et al., 1996; Rogers et al., 1999; O'Doherty et al., 2001), and optimal OFC firing rates are important for proper reversal learning to occur (Bissonette et al., 2015). Chronic ethanol exposure impairs OFC-dependent reversal learning across mice (Badanich et al., 2011; Coleman et al., 2014), rats (Brown et al., 2007; Badanich et al., 2016), primates (Jedema et al., 2011), and humans (Verdejo-García et al., 2006; Fortier et al., 2008). Previous studies in rodents and non-human primates have demonstrated that acute ethanol exposure reduces intrinsic excitability of lOFC neurons (Badanich et al., 2013; Nimitvilai et al., 2017), while ethanol dependence alters synaptic transmission and the plasticity of intrinsic excitability and blunts acute ethanol inhibition of cell firing (Nimitvilai et al., 2016, 2017; Renteria et al., 2018). In addition, lesions or chemogenetic inhibition of the lOFC increased ethanol consumption following induction of ethanol dependence (den Hartog et al., 2016), suggesting that changes in OFC cell firing may drive ethanol intake.

Like the OFC, the cingulate cortex is a key cortical region that is involved in executive control, decision-making, and reward anticipation (Chudasama et al., 2003; Stevens et al., 2011). The cingulate cortex integrates input from several limbic brain regions (Stevens et al., 2011) and functional deficits in this region are associated with impulsive drug consumption (Jentsch et al., 2014; Starski et al., 2019). A combined clinical and preclinical study showed increased glutamate levels within the cingulate cortex of patients and rats during acute withdrawal from ethanol (Hermann et al., 2012). Other studies have shown that ethanol or withdrawal can influence molecular processes and synaptic plasticity in this brain region (Li et al., 2002; Smith et al., 2017). Moreover, activation of the early immediate gene c-Fos occurs within the cingulate cortex during acute withdrawal from voluntary intermittent access to ethanol (George et al., 2012; Smith et al., 2019). Despite evidence of ethanol-induced neuroadaptations within the cingulate cortex, there is limited understanding of physiological mechanisms that drive cingulate cortex sensitivity to ethanol. Given that the lOFC and ACC play critical, yet dissociable, roles in executive function and goal-directed behavior (Sul et al., 2010; Kennerley et al., 2011), it is important to determine whether a history of voluntary consumption modifies physiological function within these regions.

Despite evidence for ethanol-induced plasticity of intrinsic excitability in multiple brain structures (Cannady et al., 2018), changes in cortical intrinsic excitability are understudied in voluntary drinking models. It is unclear how lOFC neuroadaptations develop over time and whether similar adaptations in intrinsic excitability generalize across cortical structures. Addressing these questions is important as adaptations in intrinsic excitability can facilitate synaptic integration and learning processes (Sehgal et al., 2013) and may precede drug-induced synaptic adaptations (Kourrich et al., 2015). Accordingly, an intermittent alcohol access (IAA) procedure (Rinker et al., 2017) was used to determine the time course of intrinsic excitability changes in ACC and lOFC cortical neurons from water-drinking and ethanol-drinking C57BL/6J mice. Parallel studies measured the effects of short-term and long-term ethanol consumption on adaptations in excitatory and inhibitory synaptic transmission.

## Materials and Methods

### Animals

Male C57BL/6J mice were obtained from The Jackson Laboratory (https://www.jax.org/strain/00064) at seven weeks of age. They were group-housed (four per cage) and allowed to acclimatize to the colony room for at least one week in a temperature- and humidity-controlled AAALAC-approved facility. Animals were maintained on a reverse 12/12 h light/dark cycle with lights off at 9 A.M. and had *ad libitum* access to food and water. All animals were treated in strict accordance with the *NIH Guide for the Care and Use of Laboratory Animals*, and all experimental methods were approved by the Medical University of South Carolina’s Institutional Animal Care and Use Committee.

### Two-bottle choice intermittent ethanol access

After acclimatization, mice were housed individually and were given 24-h IAA (20% v/v) and water from 9 A.M. to 9 A.M. with 24 or 48 h between drinking sessions (Mondays, Wednesdays, and Fridays; Rinker et al., 2017; Zamudio et al., 2020). Mice were subjected to the IAA model for 1 d, one week, four weeks, or seven weeks with three drinking sessions per week, and mice in the one-, four-, and seven-week groups began drinking on Wednesdays or Fridays. The location of ethanol and water bottles was alternated on each drinking session. All groups received two water bottles on intervening days. Drinking sessions were staggered so that electrophysiological recordings were performed from one mouse per recording day. Procedures were identical in age-matched control mice except mice were given access to two water bottles during drinking sessions. Mice were sacrificed 24 h following the final drinking session, and brains were extracted and prepared for whole-cell patch-clamp electrophysiology recordings. Ethanol preference was calculated from the amount of ethanol consumed as a percentage of the total amount of fluid (ethanol + water) consumed during each drinking session.

### Brain slice preparation

Brain slices containing the lOFC and ACC were prepared for whole-cell patch-clamp electrophysiology experiments from the same mouse. Following brief anesthesia with isoflurane, the brain was removed rapidly and tissue was blocked coronally for the frontal cortex. The tissue block was mounted in a Leica VT1000S vibratome containing ice-cold oxygenated (95%O_2_, 5%CO_2_) sucrose cutting solution, and coronal sections (300 µm) were cut. Slices containing the ACC or lOFC were immediately placed in a holding chamber containing oxygenated artificial CSF (aCSF) at 34°C for 30 min and kept at room temperature for at least 30 min before recordings. The composition of the cutting solution used was the following: 200 mm sucrose, 1.9 mm KCl, 1.2 mm NaH_2_PO_4_, 6 mm MgCl_2_, 0.5 mm CaCl_2_, 0.4 mm ascorbate, 10 mm glucose, and 25 mm NaHCO_3_, adjusted to 305–315 mOsm. The composition of the aCSF was the following: 125 mm NaCl, 2.5 mm KCl, 1.25 mm NaH_2_PO_4_, 1.3 mm MgCl_2_, 2.0 mm CaCl_2_, 0.4 mm ascorbate, 10 mm glucose, and 25 mm NaHCO_3_, adjusted to 290–310 mOsm. Both solutions were saturated with 95% O_2_/5% CO_2_ (pH 7.4). All reagents used to prepare aCSF, sucrose cutting solution and internal pipette solutions were purchased from Sigma.

### Whole-cell patch-clamp electrophysiology

An individual slice was placed in the recording chamber and perfused with 34°C aCSF maintained at a flow rate of 2 ml/min. Recordings were localized to deep layers of the ACC and lOFC using Zeiss Axio Examiner D1 or Olympus BX51W1 microscopes equipped with infrared Dodt gradient contrast imaging (Luigs and Neumann). Thin-wall borosilicate glass electrodes (OD = 1.5 mm, ID = 1.17 mm) were pulled on a Sutter Instrument P97 Micropipette Puller and had tip resistances ranging from 1.9 to 5.5 MΩ. Patch pipettes filled with an internal solution were slowly lowered onto the layer V pyramidal neurons to obtain a seal (>1 GΩ) followed by breakthrough to gain whole-cell access. All whole-cell recordings were conducted in large, regular spiking pyramidal neurons located in deep layers of the ACC or lOFC using Axon MultiClamp 700B amplifiers (Molecular Devices) and Instrutech ITC-18 analog-digital converters (HEKA Instruments) controlled by AxographX software (Axograph). Events were filtered at 4 kHz and digitized at a sampling rate of 10 kHz.

### Intrinsic excitability experiments

To determine the effects of IAA on the intrinsic excitability of ACC and lOFC neurons, current-clamp recordings were performed in deep-layer pyramidal neurons. Spike firing was induced by direct current injection (lOFC, 750 ms; ACC, 1000 ms) through patch pipettes filled with a potassium gluconate internal solution (120 mm KGluconate, 10 mm KCl, 10 mm HEPES, 2 mm MgCl_2_, 1 mm EGTA, 2 mm NaATP, and 0.3 mm NaGTP, adjusted to 294 mOsm, pH 7.4). All recordings were analyzed for the number of spikes in response to each current step, resting membrane potential (RMP; mV), action potential (AP) height (mV), half-width (ms), rise time (ms), and after-hyperpolarization (AHP; mV). RMP was obtained from the membrane potential just before initiating current steps. AHP magnitude was calculated by subtracting the lowest potential during hyperpolarization from AP threshold and reported values are the mean of the first three AHP magnitudes recorded. Additionally, ACC pyramidal cells were injected with hyperpolarizing current to examine potential contributions of the hyperpolarization-activated cation current (I_h_) by measuring the difference between the sag and steady-state phases of current injection (Routh et al., 2009; Salling et al., 2018). To test the effect of acute ethanol on spike firing in lOFC neurons, concentrations of ethanol (11, 33, and 66 mm) were bath applied for 8 min in a stepwise manner, followed by final washout with aCSF for at least 10 min. Cells that did not return to pre-ethanol baseline were not included in data analysis.

### Spontaneous synaptic currents

A cesium methanesulfonate internal pipette solution (125 mm CsMeSO_3_, 10 mm CsCl, 5 mm NaCl, 10 mm HEPES, 1 mm EGTA, 2 mm MgCl_2_, 5 mm MgATP, and 0.3 mm NaGTP) was used to measure spontaneous EPSCs (sEPSCs) and spontaneous IPSCs (sIPSCs) in the same neuron by recording events at a membrane potential of −70 or 10 mV, respectively (Ma et al., 2013). Each event was recorded for 5 min. Spontaneous events were detected offline using a template-matching algorithm and a threshold amplitude of 6 pA for sEPSCs and 10 pA for sIPSCs. Synaptic drive was calculated according to the following formula: sEPSC amplitude × frequency/sIPSC amplitude × frequency (Pleil et al., 2015).

### Statistical analysis

Ethanol intake and preference values are shown as box plots. Experimental data are expressed as the mean ± SEM and were analyzed with Prism software (version 8.1.0, GraphPad Software Inc.) using mixed-effects models or *t* tests, and comparisons were considered significantly different when *p *<* *0.05. All *N* values for each treatment group are shown in the figure legends, and individual values for each mouse are shown in the figures, when appropriate.

## Results

### Ethanol intake and preference

**Figure 1.**
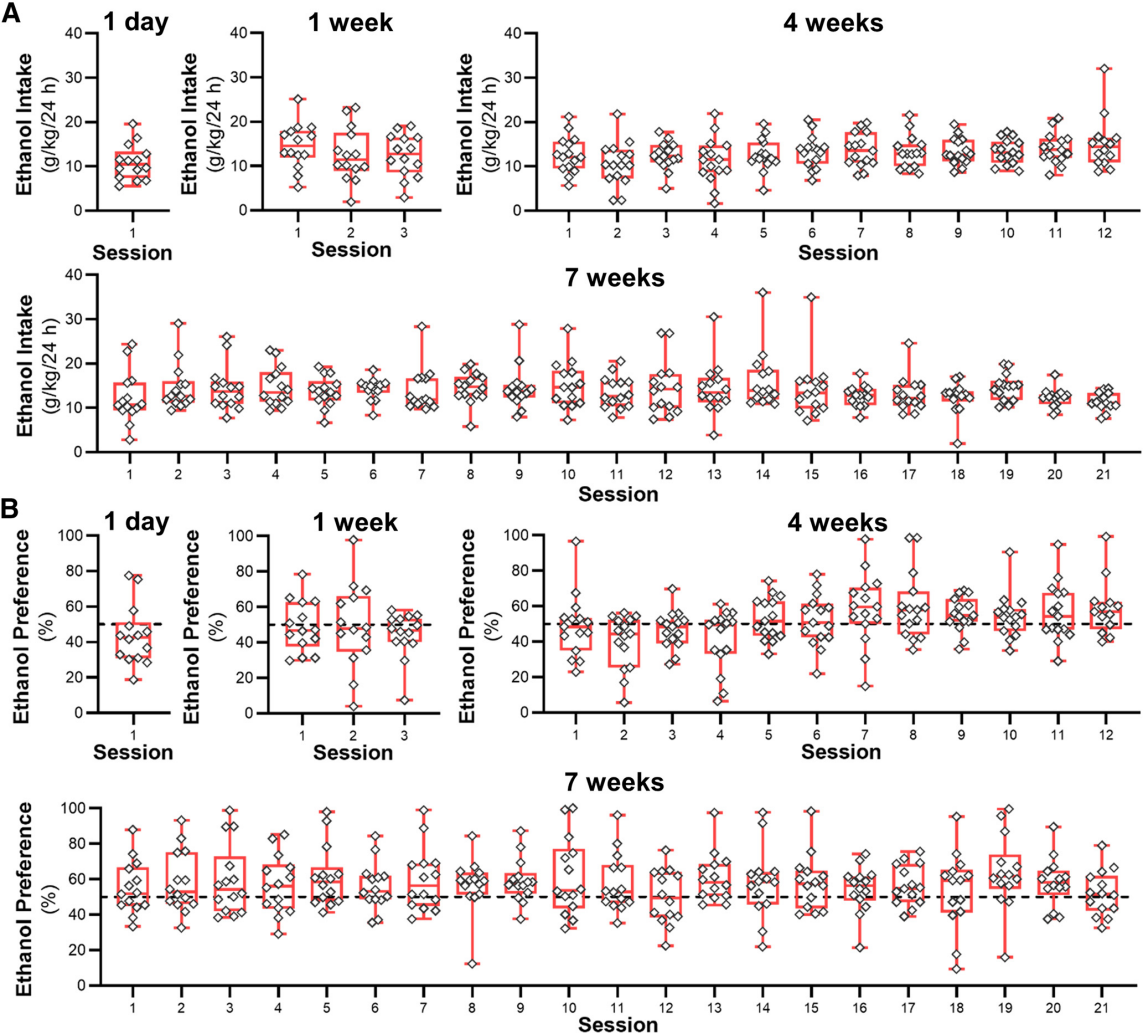
Two-bottle choice intermittent ethanol consumption and preference. ***A***, Box plots of ethanol drinking (in g/kg/24 h) from 1-d (*N* = 14 mice), one-week (*N* = 14 mice), four-week (*N* = 15 mice), and seven-week (*N* = 14 mice) groups. ***B***, Box plots of ethanol preference from 1-d, one-week, four-week, and seven-week drinking groups calculated from the amount of ethanol consumed as a percentage of the total amount of fluid (ethanol + water) consumed each drinking day.

The amount of daily ethanol (in g/kg) that mice consumed and their ethanol preference in the IAA model is shown in Figure 1. Because functional measures were obtained at four different time points throughout the model, drinking and preference data are shown for mice that were allowed to drink for 1 d, one week, four weeks, and seven weeks. There was a range of drinking and preference across individual C57BL/6J mice with daily ethanol intake averaging between 10 and 16 g/kg (Fig. 1*A*). Preference for ethanol in these mice was between 40% and 60% (Fig. 1*B*). Mice in this study did not increase their drinking across time (one week: *F*_(1.983,25.78)_ = 0.9017, *p *=* *0.417; four weeks: *F*_(5.512,76.67)_ = 1.714, *p *=* *0.1349; seven weeks: *F*_(6.111,79.44)_ = 1.143, *p *=* *0.3453), similar to that in some studies reporting no escalation of drinking when mice are given intermittent access to 20% ethanol starting on the first drinking session (Crabbe et al., 2012; Warnault et al., 2016; Rinker et al., 2017; Zamudio et al., 2020).

### Transient changes in the intrinsic excitability of ACC pyramidal neurons

**Figure 2.**
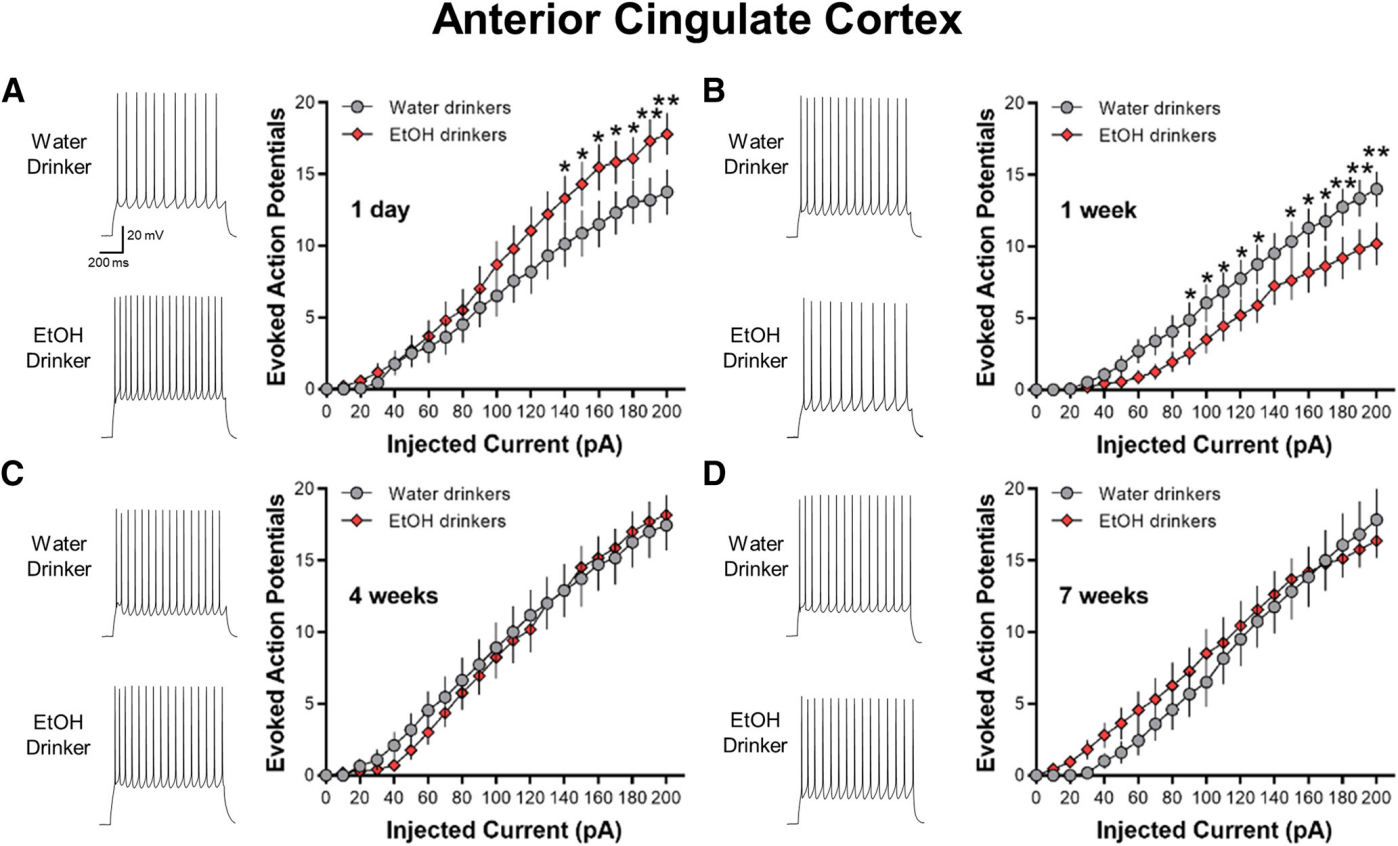
Intermittent ethanol access transiently and bidirectionally alters evoked current-induced spiking in deep-layer ACC neurons. Representative traces and number (mean ± SEM) of spikes from ACC neurons plotted against a series of 10-pA step current injections following (***A***) 1 d (*N* = 5–7 mice/group and 16–19 cells/group), (***B***) one week (*N* = 5 mice/group and 16–17 cells/group), (***C***) four weeks (*N* = 5–6 mice/group and 11–17 cells/group), and (***D***) seven weeks (*N* = 5–6 mice/group and 12–16 cells/group) of ethanol drinking. Data are expressed as the mean ± SEM plotted against a series of current injections; *p <* *0.05, **p < 0.01.

Previous studies in adult mice have demonstrated that passive ethanol vapor exposure altered intrinsic firing properties of pyramidal cells in subdivisions of the PFC (Pleil et al., 2015; Nimitvilai et al., 2016; Cannady et al., 2018). However, it is not clear whether a history of short-term or long-term voluntary ethanol consumption affects intrinsic excitability of ACC and lOFC neurons. Therefore, a series of current steps were applied to deep layer ACC pyramidal neurons of mice following IAA access to determine whether there are changes in intrinsic firing properties over the time course of ethanol consumption. Spike firing was significantly increased in ACC pyramidal cells following a single day of 24-h ethanol access (Fig. 2*A*). Statistical analysis by two-way repeated measures (RM) ANOVA with current steps as a repeating factor showed a significant increase in the number of APs in the ethanol group relative to age-matched water control mice (*F*_(30,990)_ = 1.869, *p *=* *0.0033). Interestingly, mice that consumed ethanol for one week showed reduced neuronal spiking (*F*_(30,930)_ = 2.275, *p *<* *0.001) compared with age-matched control mice (Fig. 2*B*). These bidirectional effects on ACC pyramidal cell firing appear to be transient since there were no significant differences in spike firing in mice consuming ethanol for four or seven weeks (Fig. 2*C*,*D*). Importantly, there were no significant differences in neuronal spiking between aged-matched water only mice across all time points (*F*_(60,1040)_ = 1.149, *p *=* *0.2094; Fig. 2). Other biophysical properties of ACC neurons, such as RMP, or AP threshold, height, width, or rise time, measured during current-clamp recordings were not significantly different across treatment groups (Table 1). In addition to neuronal spiking, we examined sag ratio percentage during hyperpolarizing current steps as a preliminary indicator for neuroadaptations in HCN channel function. Hyperpolarization-activated cation currents have been implicated in cellular excitability (Shah, 2014) and altered intrinsic firing properties of PFC neurons in adolescent mice following ethanol drinking (Salling et al., 2018). There were no significant differences in sag ratio percentage between ethanol-drinking and water-drinking groups across all time points (all two-tailed unpaired *t* test: *p *>* *0.05; data not shown). Taken together, these data suggest that voluntary ethanol consumption produces transient adaptations in ACC intrinsic excitability.

**Table 1.** Electrophysiological properties of ACC and lOFC deep-layer pyramidal neurons from water-drinking and ethanol-drinking mice

Drinking length	Condition	RMP(mV)	AP threshold(mV)	AP amplitude(mV)	AP width(ms)	AP rise(ms)	AHP amplitude(mV)
ACC
1 d	Water	–69.73 ± 2.18	–42.19 ± 0.90	62.25 ± 3.1	2.38 ± 0.14	0.40 ± 0.01	13.15 ± 0.77
	EtOH	–66.39 ± 1.82	–42.06 ± 0.94	70.41 ± 4.5	2.23 ± 0.10	0.43 ± 0.02	14.25 ± 0.81
1 week	Water	–69.99 ± 2.03	–41.15 ± 1.16	63.02 ± 1.8	2.20 ± 0.10	0.39 ± 0.02	15.14 ± 0.77
	EtOH	–66.46 ± 1.96	–39.50 ± 1.39	63.29 ± 2.1	1.97 ± 0.06	0.38 ± 0.01	13.79 ± 1.08
4 weeks	Water	–70.89 ± 1.81	–44.74 ± 1.43	60.80 ± 2.4	1.71 ± 0.16	0.35 ± 0.01	13.62 ± 1.03
	EtOH	–69.04 ± 2.36	–44.19 ± 1.15	57.80 ± 2.7	1.94 ± 0.13	0.38 ± 0.02	13.07 ± 0.87
7 weeks	Water	–68.70 ± 1.31	–45.68 ± 1.86	60.12 ± 2.9	2.07 ± 0.09	0.39 ± 0.02	12.29 ± 1.18
	EtOH	–70.88 ± 2.10	–44.74 ± 1.14	59.01 ± 1.9	2.33 ± 0.17	0.42 ± 0.03	13.12 ± 1.04
lOFC
1 d	Water	–68.98 ± 0.8	–38.32 ± 1.41	63.48 ± 2.07	1.82 ± 0.07	0.37 ± 0.01	16.14 ± 0.63
	EtOH	–69.47 ± 0.57	–41.89 ± 1.19	63.89 ± 1.94	1.81 ± 0.08	0.38 ± 0.01	15.19 ± 0.42
1 week	Water	–69.71 ± 0.67	–37.76 ± 1.32	64.40 ± 2.11	1.76 ± 0.04	0.36 ± 0.01	15.92 ± 0.56
	EtOH	–68.44 ± 0.64	–37.99 ± 1.49	61.49 ± 1.96	1.92 ± 0.07	0.38 ± 0.01	15.73 ± 0.67
4 weeks	Water	–69.97 ± 0.68	–37.50 ± 1.61	63.25 ± 2.15	1.98 ± 0.10	0.39 ± 0.01	16.04 ± 0.59
	EtOH	–67.87 ± 0.41*	–43.80 ± 1.38*	65.25 ± 2.13	1.73 ± 0.08	0.37 ± 0.01	13.99 ± 0.69*
7 weeks	Water	–70.07 ± 0.54	–40.29 ± 1.20	60.43 ± 2.07	1.71 ± 0.06	0.37 ± 0.01	15.17 ± 0.43
	EtOH	–69.10 ± 0.47	–41.18 ± 1.47	65.16 ± 2.29	1.81 ± 0.05	0.37 ± 0.01	15.01 ± 0.67

Values are mean ± SEM. Two-tailed unpaired *t* test was used to compare differences in electrophysiological properties between water control and ethanol drinking mice. Asterisk in bold font = *p* < 0.05 vs water drinking controls.

### IAA does not alter spontaneous synaptic transmission of ACC pyramidal neurons

**Figure 3.**
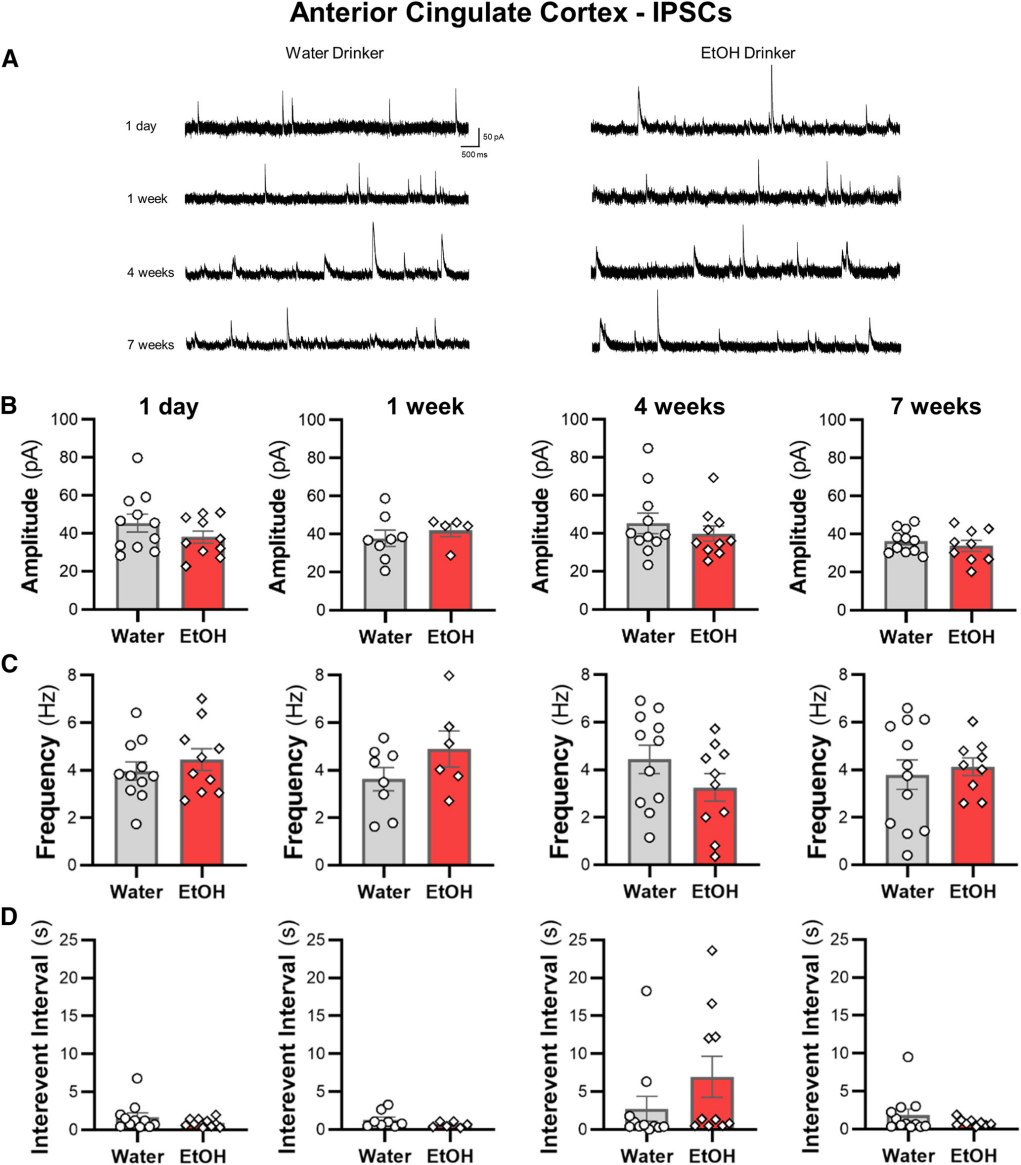
Ethanol drinking does not affect sIPSC properties in ACC pyramidal neurons. ***A***, Representative traces of sIPSCs recorded from deep-layer pyramidal neurons in the ACC from water and ethanol drinking mice across time. The (***B***) amplitude, (***C***) frequency, and (***D***) interevent interval following 1 d, one week, four weeks, and seven weeks were unaffected by ethanol drinking. White circles or diamonds represent individual values for water and ethanol drinking mice, respectively. Data are expressed as mean ± SEM; *N* = 4–5 mice/group and 6–12 cells/group.

**Figure 4.**
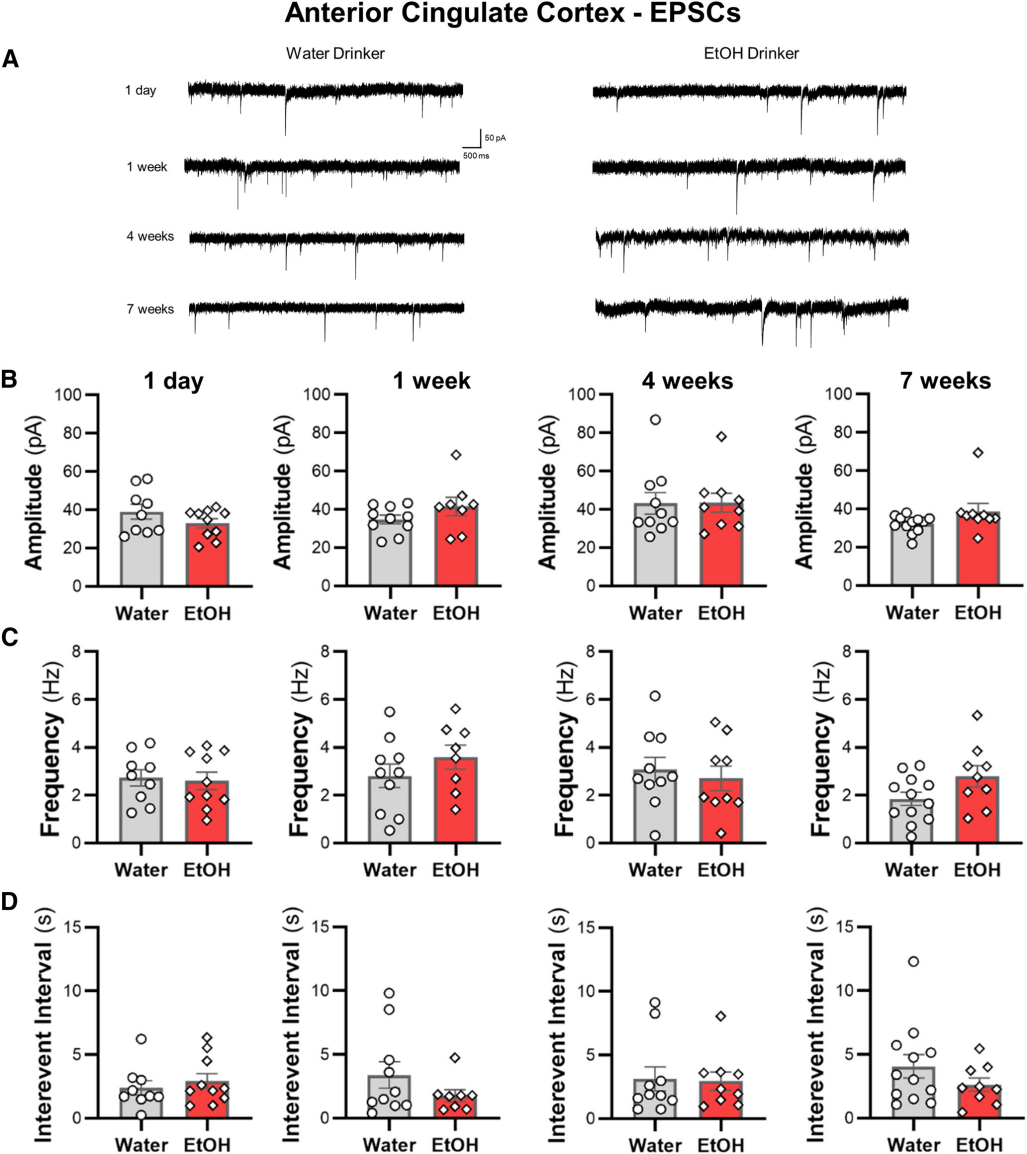
Intermittent access drinking does not affect sEPSC properties in deep-layer ACC pyramidal neurons. ***A***, Representative traces of sEPSCs recorded in ACC neurons from water and ethanol drinking mice across seven weeks of intermittent access to ethanol. The (***B***) amplitude, (***C***) frequency, and (***D***) interevent interval following a history of drinking for 1 d, one week, four weeks, and seven weeks. White circles or diamonds represent individual values for water or ethanol drinking mice, respectively. Data are expressed as mean ± SEM, *N* = 4–6 mice/group and 8–12 cells/group.

Synaptic events in the cingulate cortex are reduced following acute bath application of ethanol (Li et al., 2002). It is not clear, however, if a history of consumed ethanol alters synaptic activity of ACC pyramidal cells. EPSCs and IPSCs recorded at −70 and +10 mV, respectively, were largely unaltered by ethanol consumption in all tested drinking groups relative to water control mice (Figs. 3, 4). There were no significant effects on the amplitude or frequency of EPSCs and IPSCs in pyramidal cells of the ACC across treatment groups. In addition, integrating sEPSC and sIPSC amplitudes with frequencies to calculate an overall synaptic drive did not reveal significant differences between ethanol and water drinkers (all two-tailed unpaired *t* test: *p *>* *0.45; data not shown). These data suggest that observed changes in ACC intrinsic excitability in the drinking mice occurred independently of changes in synaptic function.

### IAA increases the intrinsic excitability of lOFC neurons

**Figure 5.**
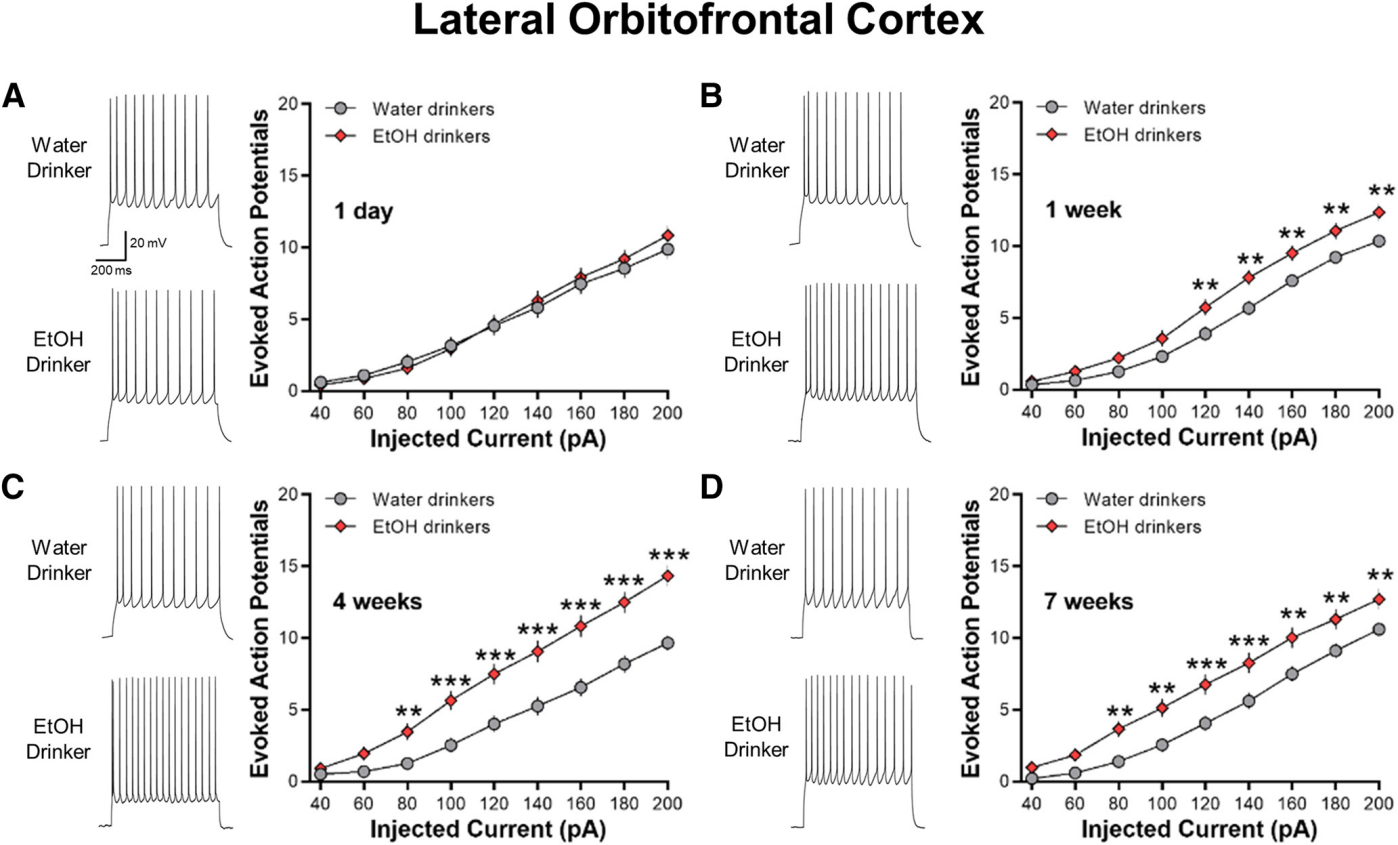
IAA enhances current-induced spiking in lOFC neurons. ***A***, Representative traces and averaged number of evoked APs showing no difference in spiking between 1-d IAA and 1-d water drinkers (*N* = 8 mice/group and 31–37 cells/group). ***B–D***, Increased evoked spiking in one-week (*N* = 8 mice/group and 36–40 cells/group), four-week (*N* = 7 mice/group and 32–33 cells/group), and seven-week (*N* = 8 mice/group and 38–39 cells/group) ethanol drinking groups as compared with their water drinking counterparts. Number (mean ± SEM) of spikes from lOFC neurons plotted against a series of current injections; **p <* *0.01, ***p <* *0.001 vs water drinking controls.

In contrast to the ACC, no significant differences in current-evoked spiking of lOFC neurons were observed between 1-d IAA mice and age-match water control mice (two-way ANOVA, *F*_(8,528)_ = 1.446, *p *=* *0.1746; Fig. 5*A*). However, lOFC AP spiking was significantly increased in one-week drinking (two-way ANOVA, *F*_(8,592)_ = 31.01, ***p <* *0.01; Fig. 5*B*), four-week drinking (two-way ANOVA, *F*_(8,504)_ = 9.755, ***p <* *0.0001; Fig. 5*C*), and seven-week drinking (two-way ANOVA, *F*_(8,600)_ = 2.764, ***p <* *0.01; Fig. 5*D*) mice as compared with age-matched water-drinking controls. Similar to the ACC, there was no age-dependent difference in AP firing across the water drinking controls (two-way ANOVA, *F*_(3,134)_ = 0.2178, *p *=* *0.889).

Similar to a previous report in ethanol-dependent mice (Nimitvilai et al., 2016), the increase in spike firing in the four-week ethanol drinking group was associated with a significant reduction in the amplitude of the AHP as compared with the four-week water drinkers (two-tailed unpaired *t* test, *t*_(62)_ = 2.254, *p =* *0.0277; Table 1). The AHP amplitudes in the 1-d, one-week, and seven-week IAA were not different from their water-drinking counterparts. Other electrophysiological characteristics of lOFC neurons obtained from water control and IAA mice are summarized in Table 1. Except for the four-week groups that also showed significant differences in the RMP (two-tailed unpaired *t* test, *t*_(63)_ = 2.652, *p =* *0.0101) and the AP threshold (two-tailed unpaired *t* test, *t*_(63)_ = 2.981, **p =* *0.0041), there were no differences in the RMP or AP threshold, height, width, or rise time between ethanol-drinking and water-drinking mice (all two-tailed unpaired *t* test: *p *>* *0.05). These results suggest that voluntary ethanol consumption increases the intrinsic excitability of lOFC neurons, similar to that observed in ethanol-dependent mice.

### Acute ethanol exposure decreases the intrinsic excitability of lOFC, except after 4 weeks

**Figure 6.**
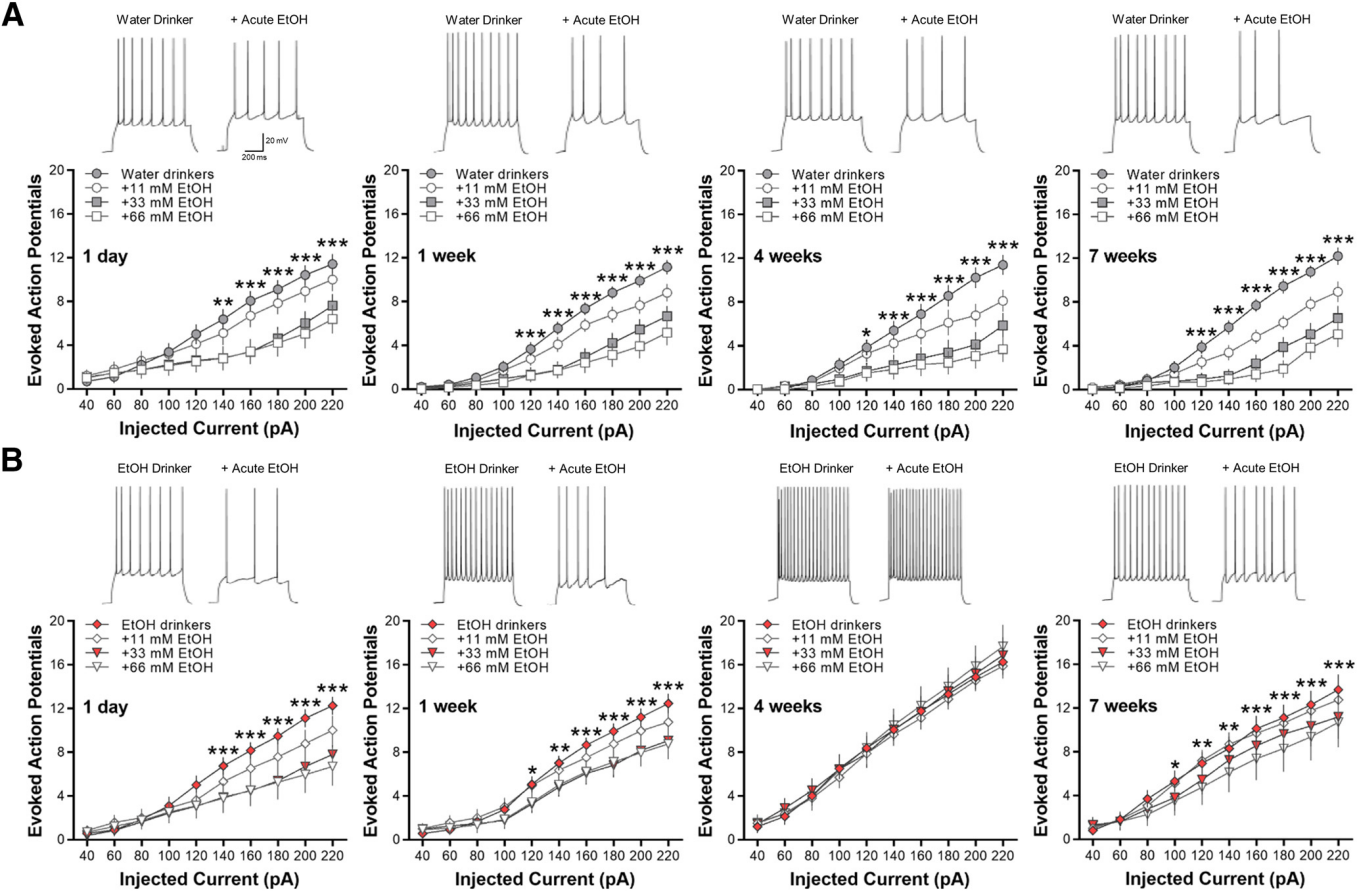
Bath application of ethanol decreases current-induced spiking of lOFC neurons in all water and ethanol drinking groups, except in mice drinking ethanol for four weeks. Representative traces showing the effects of acute ethanol (66 mm) as compared with control baseline under each drinking condition. ***A***, Ethanol significantly reduced AP spiking of lOFC neurons in all water drinking groups in a concentration-dependent manner (*N* = 5 mice/group and 16–20 cells/group). ***B***, Likewise, significant decreases in spike firing by increasing concentrations of ethanol were observed in 1-d, one-week, and seven-week drinking mice (*N* = 5 mice/group and 16–20 cells/group). In four-week ethanol drinking mice (*N* = 5 mice/group and 16 cells/group), however, acute ethanol did not affect lOFC neuron firing. Asterisks shown for 66 mm ethanol versus baseline only: *p <* *0.05, **p <* *0.01, ***p <* *0.001.

Previous studies have demonstrated that acute exposure to ethanol suppresses the intrinsic excitability of lOFC neurons in both male and female mice (Badanich et al., 2013; Nimitvilai et al., 2020) and that this inhibitory effect is lost in ethanol-dependent mice (Nimitvilai et al., 2016) and heavy drinking non-human primates (Nimitvilai et al., 2017). Here, we examined the effects of acute ethanol exposure on lOFC neurons obtained from water and ethanol drinking mice. Evoked AP spiking of lOFC neurons in all water drinking groups was reduced by bath application of ethanol in a concentration-dependent manner (two-way RM ANOVA: main effect of ethanol; *F*_(27,370)_ = 13.63, *p <* *0.0001 for 1-d water drinkers; *F*_(27,513)_ = 16.76, ***p <* *0.0001 for one-week water drinkers; *F*_(27,459)_ = 15.29, ***p <* *0.0001 for four-week water drinkers; *F*_(27,405)_ = 19.89, ***p <* *0.0001 for seven-week water drinkers; Fig. 6*A*).

Similarly, acute ethanol (11–66 mm) significantly decreased spike firing of lOFC neurons in 1-d, one-week, and seven-week ethanol drinking mice (two-way RM ANOVA: main effect of ethanol; *F*_(27,486)_ = 11.82, ***p <* *0.0001 for 1-d IAA; *F*_(27,513)_ = 8.52, ***p <* *0.0001 for one-week IAA; *F*_(27,353)_ = 5.82, **p <* *0.001 for seven-week IAA; Fig. 6*B*). In the mice that consumed ethanol for four weeks, however, there was a total loss of inhibition of lOFC neuron firing by bath application of 11–66 mm ethanol (*F*_(27,330)_ = 0.0.51, *p *=* *0.9806; Fig. 6*B*). As reported for ethanol-dependent mice and heavy drinking monkeys (Nimitvilai et al., 2016, 2017), these data demonstrate that intermittent ethanol drinking also suppresses the inhibitory effects of acute ethanol in the lOFC but only after four weeks of consumption.

### IAA does not alter spontaneous synaptic transmission of lOFC neurons

**Figure 7.**
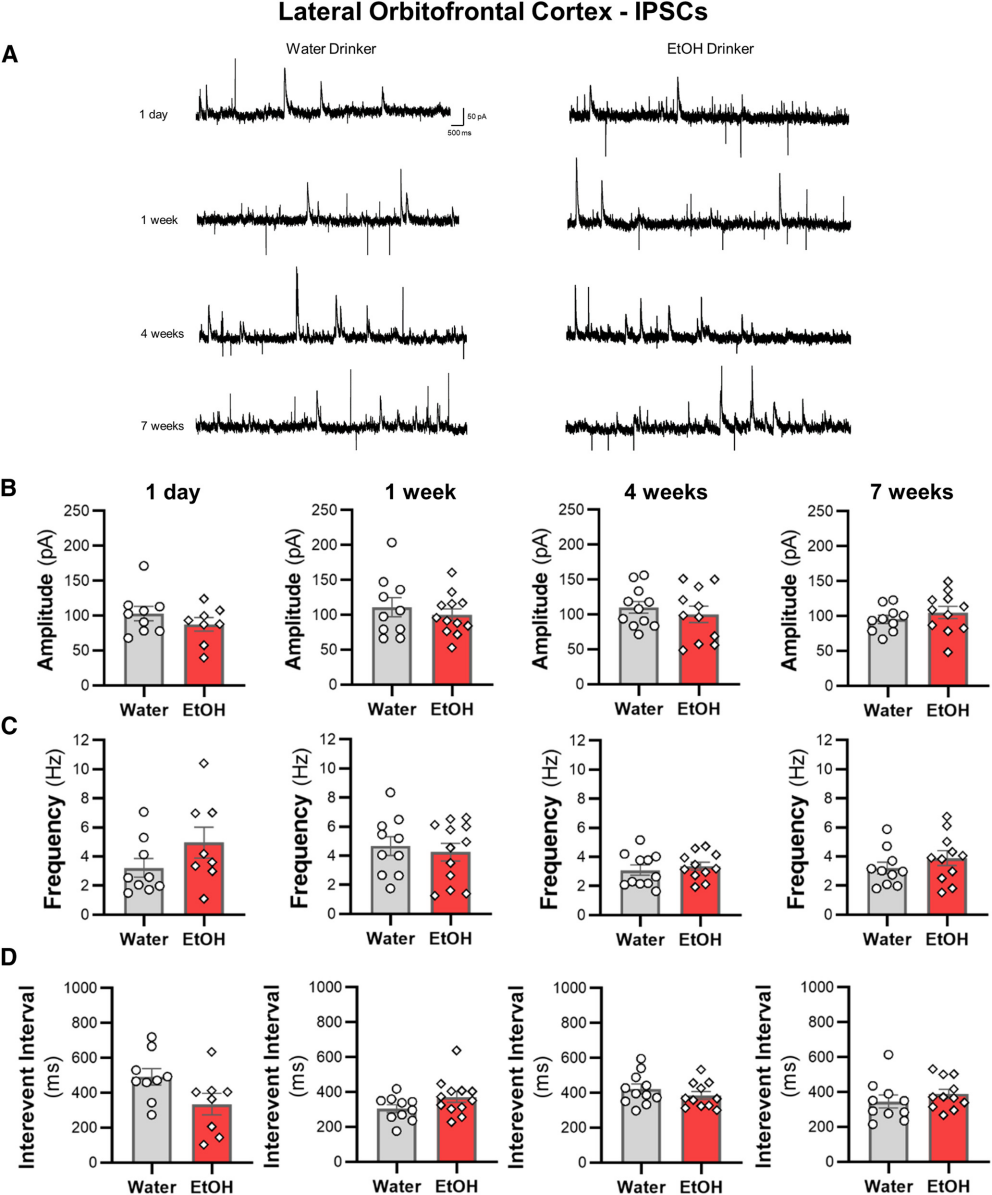
Intermittent access drinking does not affect sIPSC properties in deep-layer lOFC pyramidal neurons. ***A***, Representative traces of sIPSCs recorded in lOFC neurons from water and ethanol drinking mice across seven weeks of intermittent access to ethanol. The (***B***) amplitude, (***C***) frequency, and (***D***) interevent interval following a history of 1 d (*N* = 4 mice/group and 8–9 cells/group), one week (*N* = 4 mice/group and 10–12 cells/group), four weeks (*N* = 4 mice/group and 11 cells/group), or seven weeks (*N* = 4 mice/group and 10–11 cells/group) of ethanol drinking. White circles or diamonds represent individual values for water or ethanol drinking mice, respectively. Data are expressed as mean ± SEM.

**Figure 8.**
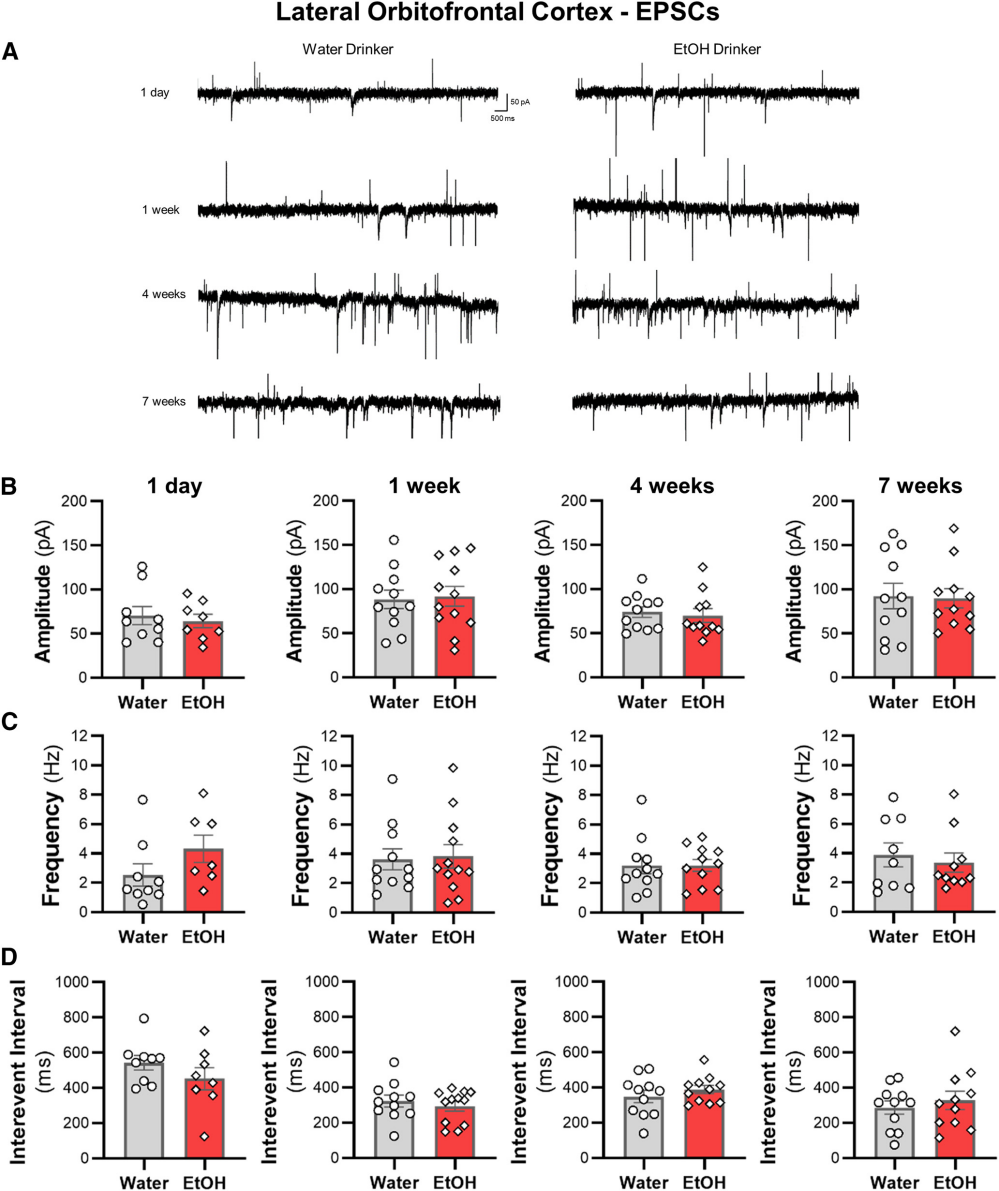
Excessive ethanol drinking does not affect sEPSC properties in lOFC pyramidal neurons. ***A***, Representative traces of sEPSCs recorded from deep-layer pyramidal neurons in the lOFC from water and ethanol drinking mice across time. The (***B***) amplitude, (***C***) frequency, and (***D***) interevent interval following a history of ethanol intake for 1 d (*N* = 4 mice/group and 8–9 cells/group), one week (*N* = 4 mice/group and 11–12 cells/group), four weeks (*N* = 4 mice/group and 11 cells/group), or seven weeks (*N* = 4 mice/group and 11 cells/group) were unaffected. White circles or diamonds represent individual values for water and ethanol drinking mice, respectively. Data are expressed as mean ± SEM.

We then examined whether sEPSCs and sIPSCs in lOFC neurons were altered across the seven weeks of ethanol drinking. There were no differences in the amplitude or the frequency of sIPSCs (Fig. 7) or sEPSCs (Fig. 8) between ethanol and water drinking mice (all two-tailed unpaired *t* test: *p *>* *0.05). Moreover, there were no shifts in synaptic drive across treatment groups (all two-tailed unpaired *t* test: *p *>* *0.05; data not shown). Consistent with the findings from the ACC, the increased intrinsic excitability of lOFC neurons following voluntary drinking can occur without functional changes in inhibitory or excitatory synaptic transmission.

## Discussion

The OFC and ACC play critical, yet dissociable, roles in executive function and goal-directed behavior (Sul et al., 2010; Kennerley et al., 2011) and work together to facilitate reinforcement-guided decision-making (Fatahi et al., 2018). The present study investigated the effects of voluntary drinking on intrinsic excitability and synaptic events in pyramidal neurons within the ACC and lOFC of mice. Furthermore, we sought to determine how drinking history affected functional plasticity within these cortical subregions. Ethanol consumption produced transient bidirectional changes in ACC intrinsic excitability that normalized after one month while changes within the lOFC were unidirectional, slower to develop, and persistent for up to seven weeks. The adaptations in the intrinsic excitability of ACC and lOFC neurons were not accompanied by significant changes in synaptic events. Thus, intrinsic mechanisms that control cell firing in these regions appear to be more sensitive to drinking-induced functional adaptations than those that regulate synaptic activity at least for the intermittent access model of voluntary ethanol intake.

Few studies have examined the contribution of the ACC to ethanol consumption, which is surprising given its important role in general fluid consumption (Gizowski and Bourque, 2018) and reward processing (Walton et al., 2006; Holec et al., 2014). To our knowledge, this study is the first to investigate how drinking history correlates to changes in ACC intrinsic firing properties. The observed transient changes in ACC excitability were interesting particularly since intrinsic excitability increased after 1 d of drinking followed by a decrease in firing after one week of drinking. The mechanisms underlying these transient changes are unclear but could reflect the encoding of reward value by the ACC during initial ethanol intake. It has been suggested that the ACC encodes the amount of effort associated with achieving a goal (Walton et al., 2006). Thus, initial responses to approach and consume the ethanol solution may have driven enhanced excitability of ACC neurons that reversed after one week of drinking when encoded memories have been consolidated. Others have reported changes in intrinsic excitability in response to other learning-mediated behaviors. For example, in rodents, mPFC intrinsic excitability was decreased after training in a response inhibition task (Hayton et al., 2011), or after a history of fear conditioning (Santini et al., 2008). Accordingly, decreases in ACC intrinsic excitability could reflect a consolidation of an ethanol-associated memory. It is also plausible that the transient change in ACC pyramidal cell spiking observed following early drinking reflect a response to novelty followed by habituation. Indeed, exposure to novelty induces activation of ACC neurons that habituates with repeated exposure to the same stimuli (Struthers et al., 2005). It is important to note that these aforementioned hypotheses are speculative due to the correlative nature of the current study, and more in-depth investigation is required to elucidate specific mechanisms that underlie changes in ACC cell firing following voluntary ethanol drinking.

With regard to the lOFC, results from the present study demonstrate that voluntary ethanol consumption enhanced the excitability of lOFC neurons, transiently reduced AHP amplitude, and suppressed the inhibition of firing by acute ethanol after four weeks of drinking. These findings are similar to previous reports that used a vapor model to generate ethanol-dependent mice (Nimitvilai et al., 2016, 2020). These data are intriguing since enhancements in lOFC intrinsic excitability emerged after one week of drinking and persisted for the duration of the study. The onset of enhanced excitability in the OFC may reflect ethanol-mediated alterations in ion channel function, such as K_Ca_2 channels that have been implicated in modulating plasticity of intrinsic excitability and reduced AHP amplitude following chronic ethanol exposure (Hopf et al., 2010; Padula et al., 2015; Nimitvilai et al., 2016). Indeed, an increase in intrinsic excitability of lOFC neurons in mice withdrawn from repeated cycles of ethanol vapor exposure was accompanied by a reduction in the AHP amplitude and a functional downregulation of apamin-sensitive K_Ca_2 channels (Nimitvilai et al., 2016). Although not tested in the present study, a decrease in the AHP amplitude of lOFC neurons in four-week IAA group could reflect a similar loss of functional K_Ca_2 channels. However, we note that the enhanced excitability of lOFC neurons observed after long-term ethanol consumption in the present study is opposite to that observed in OFC neurons from macaques with a long (more than six months) history of drinking, although those neurons also showed reduced sensitivity to acute ethanol (Nimitvilai et al., 2017), and results from a previous study in ethanol-vapor treated mice (Renteria et al., 2018). This may reflect differences in species, methodology, length of drinking history, or time point of measurement after ethanol availability. Regardless of the direction of change, these findings demonstrate that both passive and voluntary exposure to ethanol significantly alters the excitability of OFC neurons. The effects in the OFC were in contrast to the ACC where changes in intrinsic excitability emerged after a single day of drinking, demonstrating that a history of consumed ethanol differentially affects intrinsic firing of pyramidal neurons in a region-dependent and time-dependent manner. These findings add to a growing literature indicating that region-specific changes in neuronal spiking are likely the result of varying ethanol sensitivity of proteins or signaling systems that regulate cell firing.

Despite the early and robust adaptations in the plasticity of intrinsic excitability, intermittent ethanol drinking did not alter synaptic glutamatergic or GABAergic function or produce an overall change in the excitatory/inhibitory balance in the ACC or lOFC. These findings are in contrast to the enhanced synaptic plasticity reported in dopamine D1 receptor-containing medium spiny neurons in the NAc shell of mice that had access to 20% ethanol for 1 d (Beckley et al., 2016). Other models of chronic ethanol exposure, such as the ethanol vapor model, produce significant changes in synaptic transmission of lOFC neurons (Nimitvilai et al., 2016; Renteria et al., 2018) and mPFC neurons (Kroener et al., 2012; Pleil et al., 2015). In macaques, a history of chronic ethanol consumption increased the amplitude and frequency of synaptic currents and altered expression of synaptic proteins in the lOFC (Nimitvilai et al., 2017). In the present study, however, intermittent ethanol drinking did not affect the amplitude or the frequency of sEPSCs or sIPSCs in ACC and lOFC pyramidal neurons. Again, this could reflect differences in species, experimental methods, sampling times, or drinking amounts. For example, in the ethanol dependence mouse study, inhibitory and excitatory transmission was measured at 3–10 d into withdrawal (Nimitvilai et al., 2016), while in monkeys with a long history of drinking, sEPSCs were measured <12 h after the last drinking session (Nimitvilai et al., 2017). Here, we measured synaptic activity at 24 h after ethanol availability suggesting that chronic ethanol-induced changes in synaptic transmission may require longer abstinence periods or a more extensive drinking history. While evidence suggests that changes in intrinsic excitability can serve as a metaplastic mechanism to allow synaptic adaptations to occur (Sehgal et al., 2013), the results of the present study indicate that plasticity of intrinsic excitability can occur without parallel or subsequence changes in synaptic transmission following ethanol intake.

Overall, the results of the present study suggest that chronic voluntary ethanol drinking in the home cage induces transient and persistent changes in intrinsic excitability of ACC and lOFC neurons, respectively. Lack of changes in spontaneous synaptic events after IAA also suggests that alteration in synaptic transmission of ACC and lOFC neurons may vary based on the route of administration and length of abstinence. While other studies have suggested that the ACC and OFC have roles in encoding rewards, inherent limitations of the procedures used in the current study do not allow for direct measurement or comparisons of the contributions of excitability-related mechanisms to ethanol consumption. Future work using instrumental procedures combined with opto- or chemogenetic approaches that allow for more control over behavior will further elucidate the specific contributions of these brain regions in modulating the rewarding aspects of ethanol consumption. Notwithstanding the limitations of home cage drinking studies, these data provide important new insights into how voluntary ethanol drinking alters the plasticity of cortical brain regions involved in higher-order processing. These findings suggest that dynamic changes in intrinsic excitability of cortical neurons could contribute to cognitive dysfunction and excessive drinking observed in individuals with AUD.

## Synthesis

Reviewing Editor: Karen Szumlinski, University of California at Santa Barbara Decisions are customarily a result of the Reviewing Editor and the peer reviewers coming together and discussing their recommendations until a consensus is reached. When revisions are invited, a fact-based synthesis statement explaining their decision and outlining what is needed to prepare a revision will be listed below. The following reviewer(s) agreed to reveal their identity: NONE.

I would like to begin by apologizing for the uncharacteristically long time that I took to assemble the reviews of your article for eNeuro. I have now had the time to review the comments by our two expert reviewers and we have come to the decision of “Revise and Re-review” as the work required should take less than 2 months to revise. Now, with the current Covid-19 shut-down, eNeuro understands that you may not be able to perform the experiments in a timely manner and if you are unable to complete the revisions in a 2-month period, you can simply contact the eNeuro office and ask for an extension. We are very understanding of the situation and would be more than happy to assist you in any manner possible.

Both reviewers considered the premise of the report to be excellent - the study of neuroplastic changes that occur with extended-access alcohol consumption is a timely research topic. However, both reviewers had issues with the interpretation of the data due to the unfortunate fact that the subjects in your report did not escalate their intake during the drinking procedures. Both reviewers also raised the issue that the analyses were purely correlational and partially time-dependent, which reduces the overall significance of the findings. Please find below the specific comments of each reviewer. I hope you find these comments helpful as you move forward revising your report for eNeuro.

Reviewer 1:

In this manuscript the authors investigate the impact of short or long (up to 7 weeks)) voluntary alcohol drinking on the biophysical properties and synaptic inputs to neurons in the lateral orbitofrontal cortex (lOFC) and anterior cingulate cortex (ACC) in mice. The lOFC and ACC play important roles in executive functions and impulse control, and alcohol-induced cognitive deficits may contribute to an escalation of drinking and the transition to alcohol dependence. Therefore, this paper asks an important question as to how voluntary alcohol consumption affects the function of the principal cells in these two brain regions in a time-dependent manner. To answer this question, the authors use an intermittent alcohol access (IAA) procedure to expose mice to alcohol for various durations (1 day, 1 week, 4 weeks, or 7 weeks) and they then measure changes in action potential firing, intrinsic membrane properties, and synaptic transmission (measured as spontaneous excitatory and inhibitory currents). They observe a transient decrease in action potential firing in ACC pyramidal cells that is evident only after 1 week, but disappears by week 4. Changes in excitability were not accompanied by changes in synaptic transmission. In contrast, pyramidal cells of the lOFC showed persistent (up to 7 weeks) increases in action potential firing after 1 week of IAA, but also no changes in synaptic transmission. The changes in AP firing were not consistently associated with changes in intrinsic membrane properties.

The design of the experiments is straightforward, and the organization of the manuscript and description of the data is clear. The analysis of the data is adequate and the figures illustrate the main point of the analysis well. The paper is well written, although the discussion tends to overinterpret the available data in some points.

A weakness of the study is that all experiments are purely correlational and that the functional relevance of the observed changes is therefore difficult to gauge. This is somewhat compounded by the fact that the authors measured a large number of different parameters in two different brain regions, and while some measures increased persistently others changed in a transient manner, while yet others remained completely unchanged under these conditions.

Major

The mice show an increase in EtOH consumption after 1 week of IAA, but no escalation of intake from day 1 over 4 or 7 weeks. Why do the authors think this is? Or rather, why do they think that no escalation occurred as has consistently been reported in the past?

IAA increases the intrinsic excitability of lOFC neurons in all but the 1-day drinking group; however, it only affects the response to acute EtOH application in the 4-week group. The effect seems very robust across a range of EtOH concentrations (Figure 6B); however, it begs the question what is special about the 4-week timepoint? Because of the many variables and time points analyzed, the study runs the risk of type 1 errors. This becomes problematic when in lines 266-268 the authors state “[...] that intermittent ethanol drinking also suppresses the inhibitory effects of acute ethanol in the lOFC.” In fact, even when not counting the 1-day EtOH drinking condition, IAA only suppressed the acute effects of EtOH in 1 out of 3 conditions.

Because the results are strictly correlational the discussion overreaches in parts, for example in the interpretation of the time-dependent changes in AP firing in the ACC.

Minor

It would be very helpful to further clarify the procedures for IAA. In line 98 of the Methods sections the authors state that the period between drinking sessions was 24 or 48 hrs. It should be stated which of the 2 time periods applied to which drinking groups since this would make a big difference in the number of exposures, particularly for the 1- and 4-week drinking groups.

Line 184 refers to the longest drinking period as 12 weeks; however, in all figures the longest duration is 7 weeks.

Typos

Line 34: “impede(s)"

Line 103: “accept” should probably be “except"

Line 116: delete the comma

Line 199: 3 weeks or 1 week?

Reviewer 2:

This report employs an intermittent alcohol access paradigm. Typically, the IA procedure leads to increased drinking and preference. However, in this study, IA for some reason does not. While others have also failed to detect an increase in drinking and preference with IA procedures (including my own laboratory), the fact that the drinking did not escalate presents a problem for interpretation.

The authors demonstrate changes in excitability, consistent with changes in SK function, which would be a finding consistent with other reports by the Mulholland laboratory. However, the authors do not don't really discuss this possibility or evaluate this idea.

The authors discuss Ih, but no data pertaining to Ih is provided. The gold standard for measuring Ih is a current blocked by ZD7288 (e.g., Salling et al.). For some reason, their pyramidal cells look more excitable than what is typically observed in the literature and do not appear to accommodate rapdily during depolarization. This is not discussed.
